# Type-Strain-Anchored Phylogenomics Reveals Taxonomic Discordance and Functional Variation in *Agrobacterium*

**DOI:** 10.3390/ijms27209210

**Published:** 2026-10-16

**Authors:** Maria S. Filatova, Peter V. Evseev, Alexander N. Ignatov, Polina V. Asmaeva, Andrey V. Chaplin, Konstantin A. Miroshnikov

**Affiliations:** 1 Shemyakin-Ovchinnikov Institute of Bioorganic Chemistry, Russian Academy of Sciences, Miklukho-Maklaya Str. 16/10, 117997 Moscow, Russia; filat00va.maria@gmail.com; 2 Nelyubin Institute of Pharmacy, Sechenov First Moscow State Medical University ( Sechenov University), Trubetskaya Str. 8, bldg. 2, 119991 Moscow, Russia; 3 Pirogov Russian National Research Medical University, Ostrovityanova Str. 1, 117997 Moscow, Russia; asmaeva19@gmail.com (P.V.A.); okolomedik@gmail.com (A.V.C.); 4 Agrobiotechnology Department, Agrarian and Technological Institute, RUDN University, Miklukho-Maklaya Str. 6, 117198 Moscow, Russia; an.ignatov@gmail.com

**Keywords:** *Agrobacterium*, bacterial taxonomy, phylogenomics, *Agrobacterium tumefaciens* complex

## Abstract

Species names deposited for *Agrobacterium* genomes do not always correspond to genomic lineages, complicating taxonomy and functional comparisons. We analyzed 432 genome records, including 400 assigned to *Agrobacterium*, using ANI, AAI, type-strain dDDH comparisons, and phylogenies based on 120 bacterial markers and 42 ribosomal proteins. ANI clustering at 95% identified 29 *Agrobacterium* groups, including five highlighted candidate species-level lineages. Both phylogenies supported a genus-level clade, although one ANI group showed discordance between the ribosomal-protein ML topology and bootstrap consensus. Type-strain dDDH supported conspecificity of *A. fabacearum* and *A. tumefaciens* (86.0%), whereas *A. leguminum* and *A. deltae* were below the conventional species boundary (66.7%). Pan-genome analysis of 31 representatives revealed extensive gene-content variation. Across 400 *Agrobacterium* records, 148 of 342 KEGG modules reached the reconstruction threshold in at least one genome, and 58 did so in all genomes. In the full 432-record dataset, 174 genomes had Ti-associated and seven had Ri-associated marker profiles; *catB1* and *tet(30)* were detected in 243 and 29 genomes, respectively. These results identify taxonomic assignments requiring reassessment and link genomic grouping to variation in predicted functional repertoires.

## 1. Introduction

Early classifications of *Agrobacterium* placed considerable weight on plant disease phenotypes. However, by the 1970s, physiological and biochemical differences had established a biotype-based classification, and Kerr and Panagopoulos (1977) distinguished biotypes 1, 2, and 3 [1]. This classification preceded the characterization of Ri plasmids and was distinct from the classification of strains by disease phenotype. Subsequent studies applied this biotype framework to strain characterization [2]. Crown gall and hairy root disease are associated with tumor-inducing (Ti) and root-inducing (Ri) plasmids, respectively. Because virulence plasmids occur in different genomic backgrounds, pathogenicity alone is insufficient for species identification [3].

The boundaries of the genus have also been disputed. The proposal to include *Agrobacterium* in *Rhizobium* was followed by multilocus and phylogenomic studies supporting a revised circumscription of *Agrobacterium* within *Rhizobiaceae* [4,5,6]. At the species level, DNA–DNA hybridization and amplified fragment length polymorphism analyses distinguished multiple genomic species within *Agrobacterium* biovar 1, traditionally associated with the *A. tumefaciens* species complex. Several of these genomic species could not be distinguished using conventional biochemical tests [7]. The species complex is therefore broader than *A. tumefaciens sensu stricto*, the species containing its nomenclatural type strain. The present study also takes *Agrobacterium* lineages outside this complex and neighboring genera into consideration.

Single-gene phylogenies based on 16S rRNA or *recA* do not reliably resolve all genomospecies in the complex [8]. Genome-wide comparisons provide greater resolution, with average nucleotide identity (ANI) values around 95–96% commonly used as a reference range for bacterial species delimitation [9]. At the genus level, core-genome phylogeny and core-proteome amino acid identity have been used together to reassess boundaries within *Rhizobiaceae* [10].

Previous genome-based studies have identified misclassified strains and questioned the separation of some officially named species [8]. These are different taxonomic problems: a deposited genome may carry an incorrect species name, while two named species may themselves represent the same genomic lineage (i.e., one name may be a later synonym of the other). Evaluating either case requires comparisons with the corresponding nomenclatural type strain and the type strains of closely related species [11]. This also affects the interpretation of functional diversity. Grouping genomes by deposited names alone can confound variation within a species with differences between species. A consistent reference for both taxonomic assessment and functional comparison is therefore the correspondence between names, type strains, and genomic lineages.

Functional differences have already been linked to particular lineages within the complex. Lassalle et al. identified genes associated with genomovar G8, the “*A. fabrum*” lineage, and experimentally verified functions including ferulic acid degradation and curdlan production. Their findings supported a hypothesis of ecological differentiation [12]. This evidence raises a question for comparisons across the genus: which functions are broadly conserved, which are associated with genomic lineages, and which vary among members of the same lineage? Addressing this question requires gene-content and metabolic comparisons to use the same taxonomic assignments as the phylogenomic analysis. A related question concerns the distribution and evolution of both virulence plasmids and other accessory megaplasmids, and their relationships with chromosomal lineages. Here, we address the Ti/Ri-associated component through marker screening; other accessory replicons are discussed as an important extension requiring replicon-resolved comparisons [13].

In this study, we reassessed species boundaries and the phylogenetic coherence of *Agrobacterium* using genomes from the genus and closely related taxa. ANI and AAI comparisons were combined with phylogenies based on 120 conserved bacterial markers and 42 ribosomal proteins. Nomenclatural type strains anchored the interpretation of genomic groups, and dDDH comparisons addressed disputed species relationships. Pan-genome and KEGG (Kyoto Encyclopedia of Genes and Genomes) module analyses were then used to examine gene-content variation and predicted metabolic potential, alongside Ti/Ri plasmid-associated signatures and antibiotic resistance-related genes. The study links reassessment of deposited identifications and unresolved species boundaries to the distribution of predicted functional repertoires within and between genomic lineages.

## 2. Results

### 2.1. ANI Clustering and Species-Level Relationships

**Figure 1:**
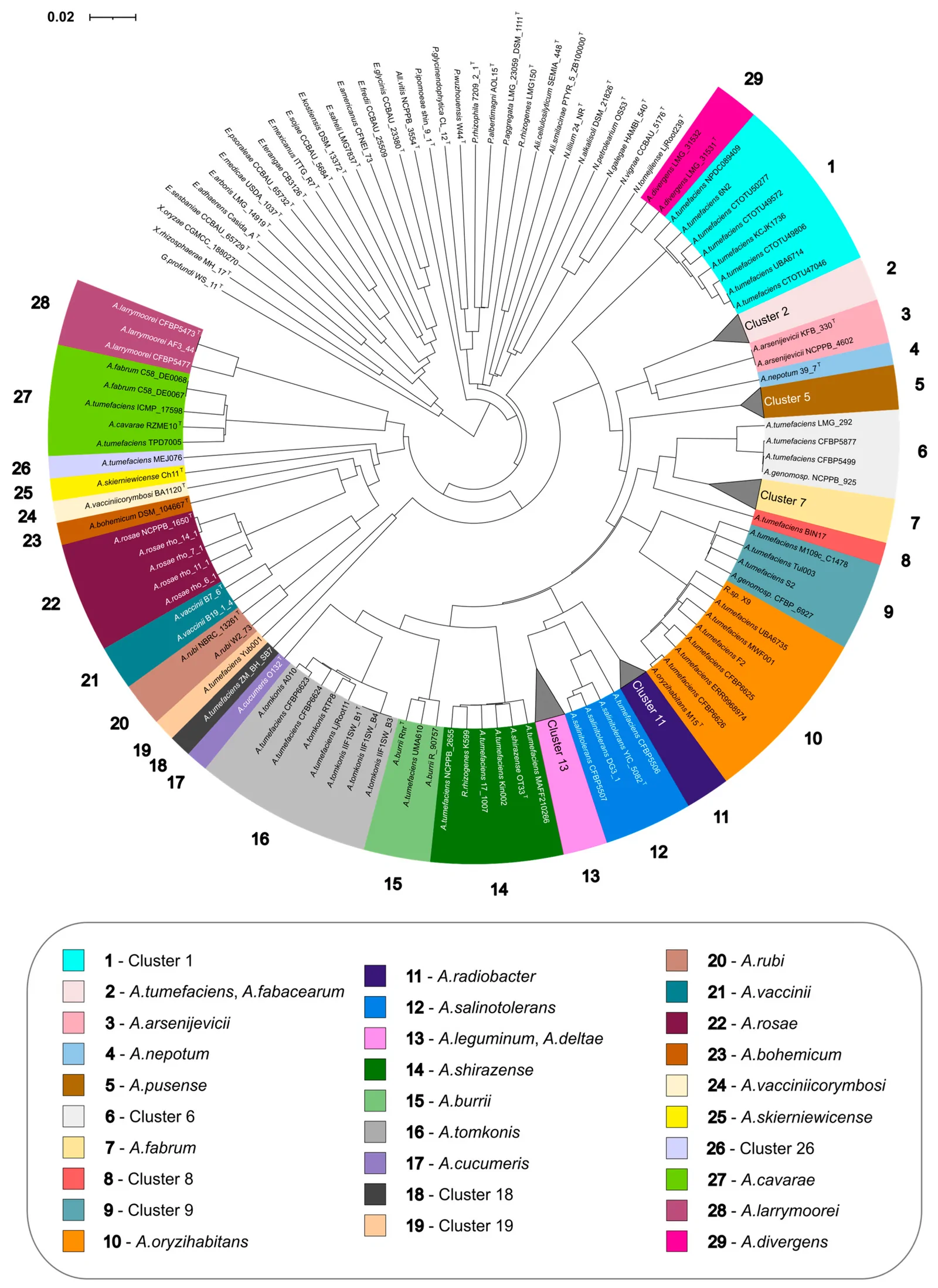
UPGMA dendrogram constructed from pairwise ANI values for 432 genomes of the *Agrobacterium* complex and closely related taxa. Numbers indicate cluster designations at the 95% ANI threshold.

ANI clustering was evaluated at 95% and 96%, within the reference range commonly used for bacterial species delimitation [9,14]. The analysis included 432 genome records from *Agrobacterium* and related taxa; ungrouped genomes were retained as singletons. Figure 1 shows the dendrogram at 95%, and Supplementary Table S1 compares cluster membership at both thresholds.

The full dataset yielded 61 clusters at 95% ANI and 67 at 96%, including singletons. Of the clusters at 95%, 29 belonged to *Agrobacterium* (Table 1). Cluster 13 contained 56 genome records, including the type strains *A. leguminum* MOPV5^T^ and *A. deltae* YIC 4121^T^, and was split into three clusters at 96%. The two type strains occurred in separate derived clusters, while the third contained no type strain. By contrast, Cluster 2, containing 120 records and the type strains *A. tumefaciens* ATCC 4720^T^ and *A. fabacearum* CNPSo 675^T^, retained the same membership at both thresholds.

Two other type-strain-containing clusters split at 96% ANI. *A. rubi* NBRC 13261^T^ and W2/73 grouped together at 95% but separated at 96%; the same pattern was observed for *A. vaccinii* B7.6^T^ and B19.1.4.

Five clusters were highlighted as unresolved candidate species-level lineages: Cluster 1, containing eight genomes deposited as *A. tumefaciens*, and the singletons BIN17, ZM-BH-SB7, Yub001, and MEJ076 (Clusters 8, 18, 19, and 26, respectively). Cluster 1 overlaps candidate genomospecies G21, and BIN17 overlaps candidate G24, as identified by Vargas Ribera et al. [8]. These five groups are distinct from clusters containing deposited genomospecies designations or reference strains of previously proposed species and merit further taxonomic evaluation.

Genomes deposited as *A. tumefaciens* were distributed across several ANI clusters, including clusters containing the type strains of *A. radiobacter* and *A. pusense* and the “*A. fabrum*”/G8 lineage. Thus, the deposited name *A. tumefaciens* did not correspond to a single ANI-defined group.

**Figure 2:**
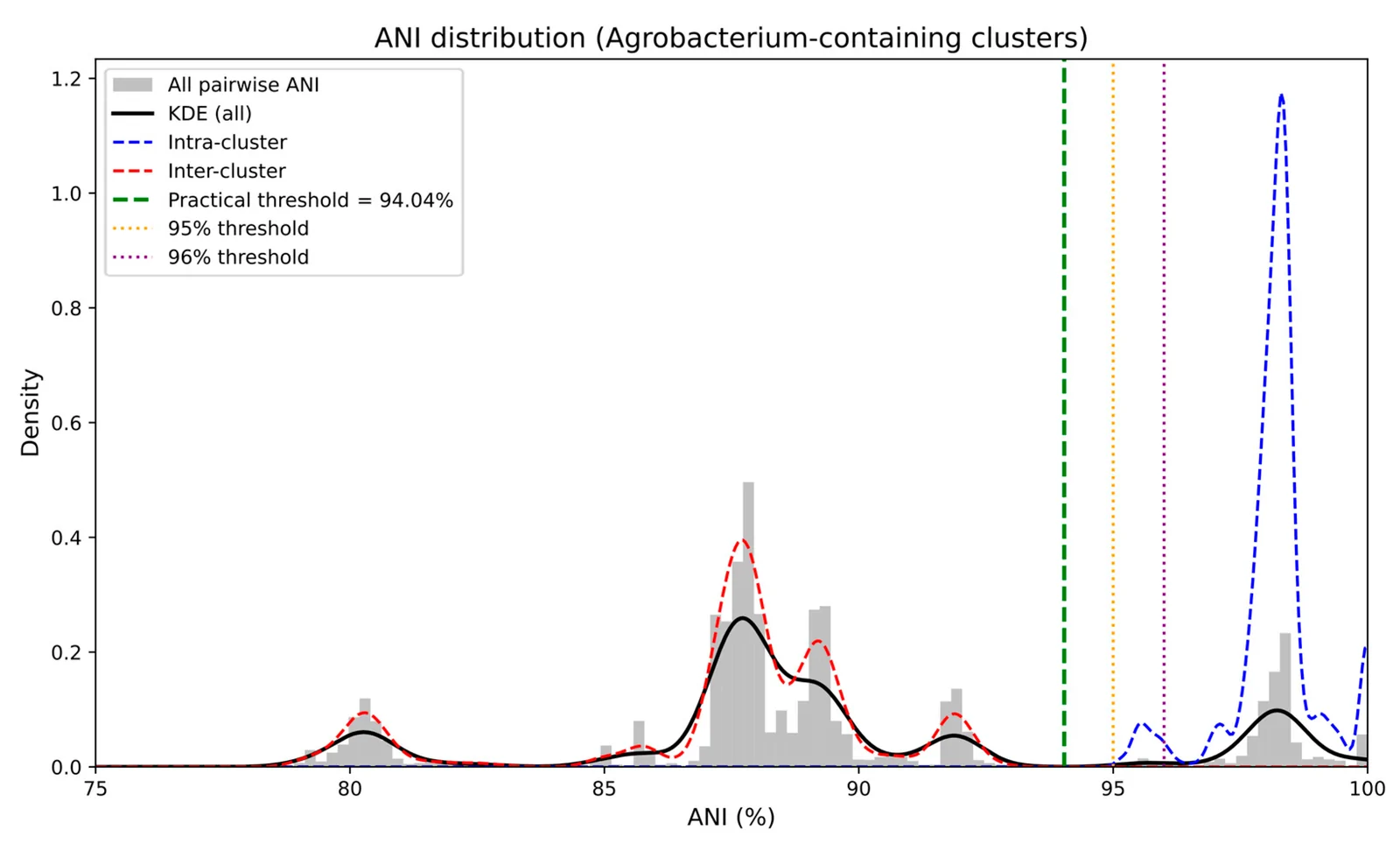
Distribution of pairwise ANI values for 400 *Agrobacterium* genome records. Gray bars show all comparisons; the black curve shows the overall KDE, and blue and red dashed curves show within-cluster and between-cluster KDEs, respectively. The green line marks the estimated crossover of KDEs (94.04%); orange and purple lines indicate 95% and 96% ANI.

The distribution of pairwise ANI values was bimodal, with the estimated crossover between the plotted comparison categories at approximately 94% (Figure 2). The 95% and 96% thresholds used for clustering are shown for reference.

The full ANI heatmap and UPGMA dendrogram at 95% are provided in Supplementary Figure S1.

**Figure 3:**
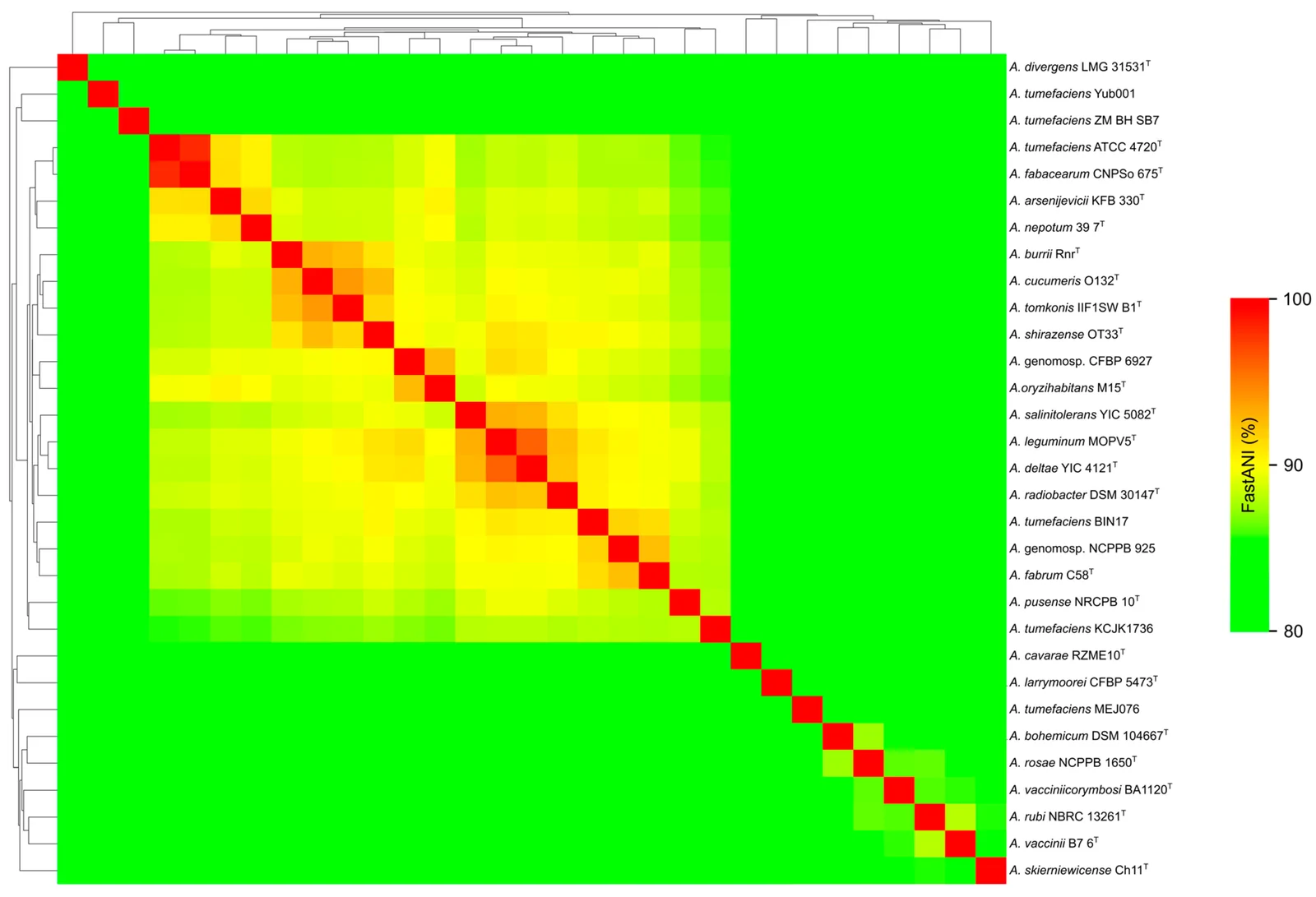
ANI heatmap for representative genomes from *Agrobacterium* ANI clusters at the 95% threshold.

The representative-genome ANI heatmap provides a compact comparison of the ANI-defined groups, including the putative species-level lineages (Figure 3). Type strains were used as representatives where available.

Selected species-level relationships were additionally evaluated using dDDH between type strains and selected non-type strains (Supplementary Table S2). Using GGDC formula 2 (GBDP d4), the type strains of *A. tumefaciens* ATCC 4720^T^ and *A. fabacearum* CNPSo 675^T^ showed a dDDH value of 86.0% (95% CI, 83.4–88.3%), above the conventional 70% species threshold. In contrast, *A. leguminum* MOPV5^T^ and *A. deltae* YIC 4121^T^ showed a dDDH value of 66.7% (95% CI, 63.7–69.5%). Comparisons of *A. tumefaciens* BIN17 and *A. radiobacter* DSM 30147^T^ with these two type strains yielded substantially lower dDDH estimates obtained using formula 2/d4, ranging from 38.5% to 45.1% (Supplementary Table S2).

### 2.2. AAI Clustering and Genus-Level Relationships

AAI cluster membership was compared across seven thresholds from 65% to 79% (Supplementary Table S3).

At 74%, 75%, and 76%, all 400 *Agrobacterium* genome records remain combined in one cluster. At 76%, the included *Aliirhizobium* and *Neorhizobium* representatives separated from each other. This was the highest tested threshold retaining the complete *Agrobacterium* set in one cluster, and it was used as the operational cutoff for presentation. A similar full-proteome AAI value was reported in a recent analysis of agrobacteria and rhizobia [15].

At 77%, *A. divergens* LMG 31531^T^ and LMG 31532 separated from the remaining 398 *Agrobacterium* genomes, and the same 398 + 2 partition was retained at 78%. At 79%, additional *Agrobacterium* lineages separated, yielding a 377 + 21 + 2 partition. Thus, intrageneric fragmentation began above 76%, whereas 76% represented the most stringent tested cutoff that retained all sampled *Agrobacterium* genomes in a single cluster.

**Figure 4:**
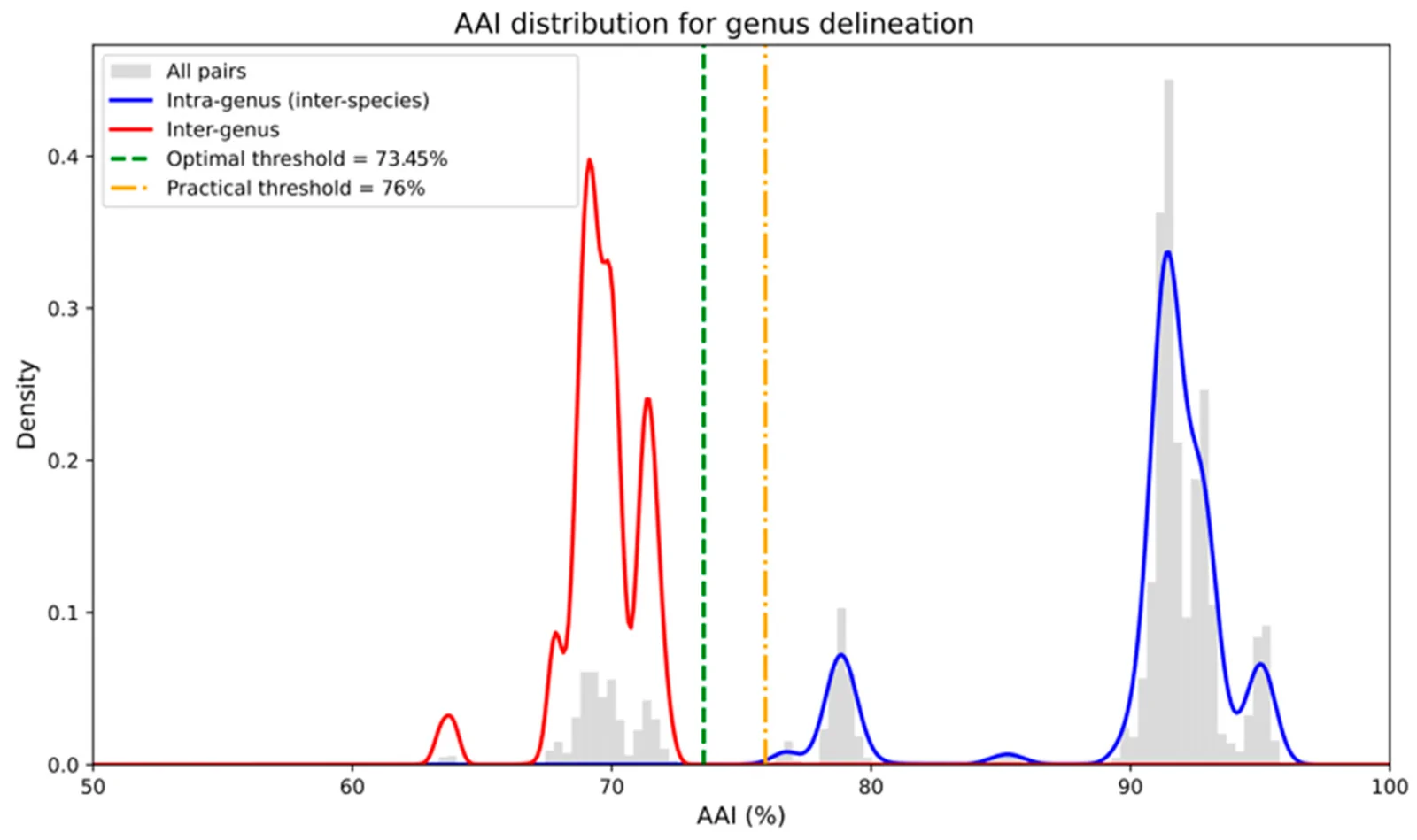
Distribution of pairwise AAI values. Gray bars show all comparisons. Blue and red curves show KDEs for the plotted intra-genus (inter-species) and inter-genus categories, respectively. The green line marks the estimated crossover (73.45%); the orange line indicates the dataset-specific operational cutoff of 76%.

**Figure 5:**
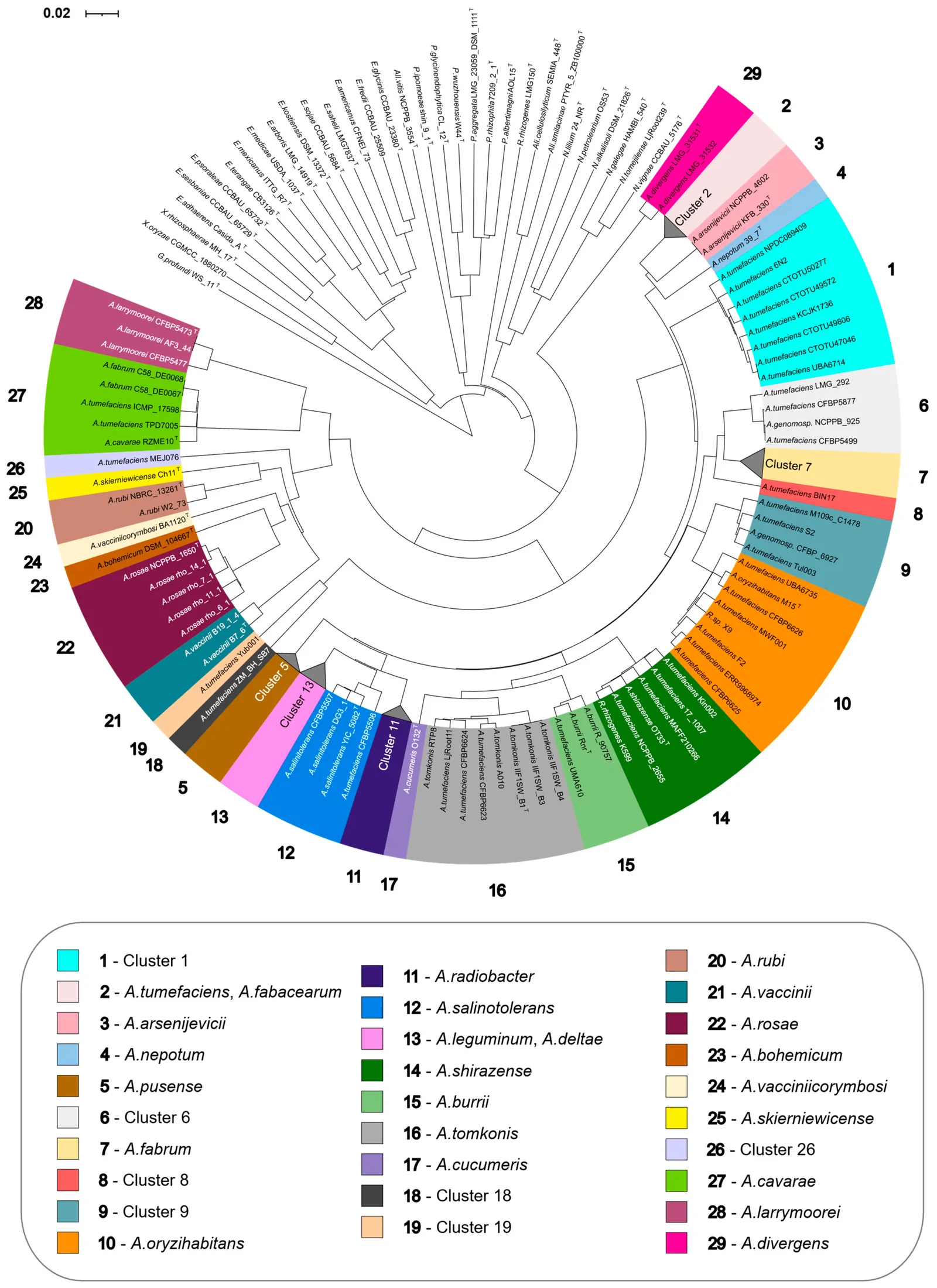
UPGMA dendrogram based on pairwise AAI values for 432 genomes of the *Agrobacterium* complex and related taxa. Colored sectors and numbers denote ANI clusters defined at the 95% ANI cutoff and are overlaid to show their distribution on the AAI dendrogram. At the 76% AAI cutoff, all genomes assigned to *Agrobacterium* formed a single cluster.

The plotted intra-genus and inter-genus AAI distributions intersected at 73.45% (Figure 4). This descriptive crossover differed from the 76% operational clustering cutoff. The AAI dendrogram is shown in Figure 5, and the full heatmap in Supplementary Figure S2.

### 2.3. Phylogeny Inferred from GTDB-Tk Markers

The concatenated alignment of 120 bacterial marker proteins comprised 432 genome records and 5010 amino acid positions. Maximum-likelihood inference was performed with IQ-TREE 3.0.1; ModelFinder selected Q.INSECT+F+I+R4 as the best-fit model under the Bayesian information criterion (BIC). The model included empirical amino acid frequencies, an estimated proportion of invariable sites of 63.07%, and four FreeRate categories for the variable sites. Branch support was assessed with 1000 ultrafast bootstrap replicates. The maximum-likelihood tree had a log-likelihood of −31,364.36. The bootstrap consensus tree had a log-likelihood of −31,368.15 and a Robinson–Foulds distance of 76 from the ML topology. IQ-TREE flagged 141 near-zero internal branches (<0.0002 substitutions per site), which were treated as poorly resolved.

All genomes assigned to *Agrobacterium* formed a clade with 100% ultrafast bootstrap support. The tree also separated the included *Rhizobium*, *Xaviernesmea*, and *Sinorhizobium*/*Ensifer* lineages. *Allorhizobium vitis* occurred outside the *Agrobacterium* clade. *Georhizobium profundi* WS-11^T^ was used as the outgroup.

Within *Agrobacterium*, a subclade with 100% support contained representatives of *A. skierniewicense*, *A. larrymoorei*, *A. rubi*, *A. rosae*, *A. vaccinii*, *A. cavarae*, “*A. bohemicum*”, and *A. vacciniicorymbosi*. This subclade also encompassed three genomes deposited as *A. tumefaciens*: TPD7005 and ICMP 17598 grouped with *A. cavarae* RZME10^T^, whereas MEJ076 formed a separate lineage.

The “*A. fabrum*”/G8 and *A. pusense* lineages each received 100% support. *A. tumefaciens* ATCC 4720^T^ and *A. fabacearum* CNPSo 675^T^ occurred in the same broader clade (95% support). *A. radiobacter* DSM 30147^T^ grouped with genomes deposited as *A. radiobacter* and *A. tumefaciens* in ANI Cluster 11. Other genomes deposited as *A. radiobacter* occurred in the group containing *A. leguminum* MOPV5^T^ and *A. deltae* YIC 4121^T^ (ANI Cluster 13).

**Figure 6:**
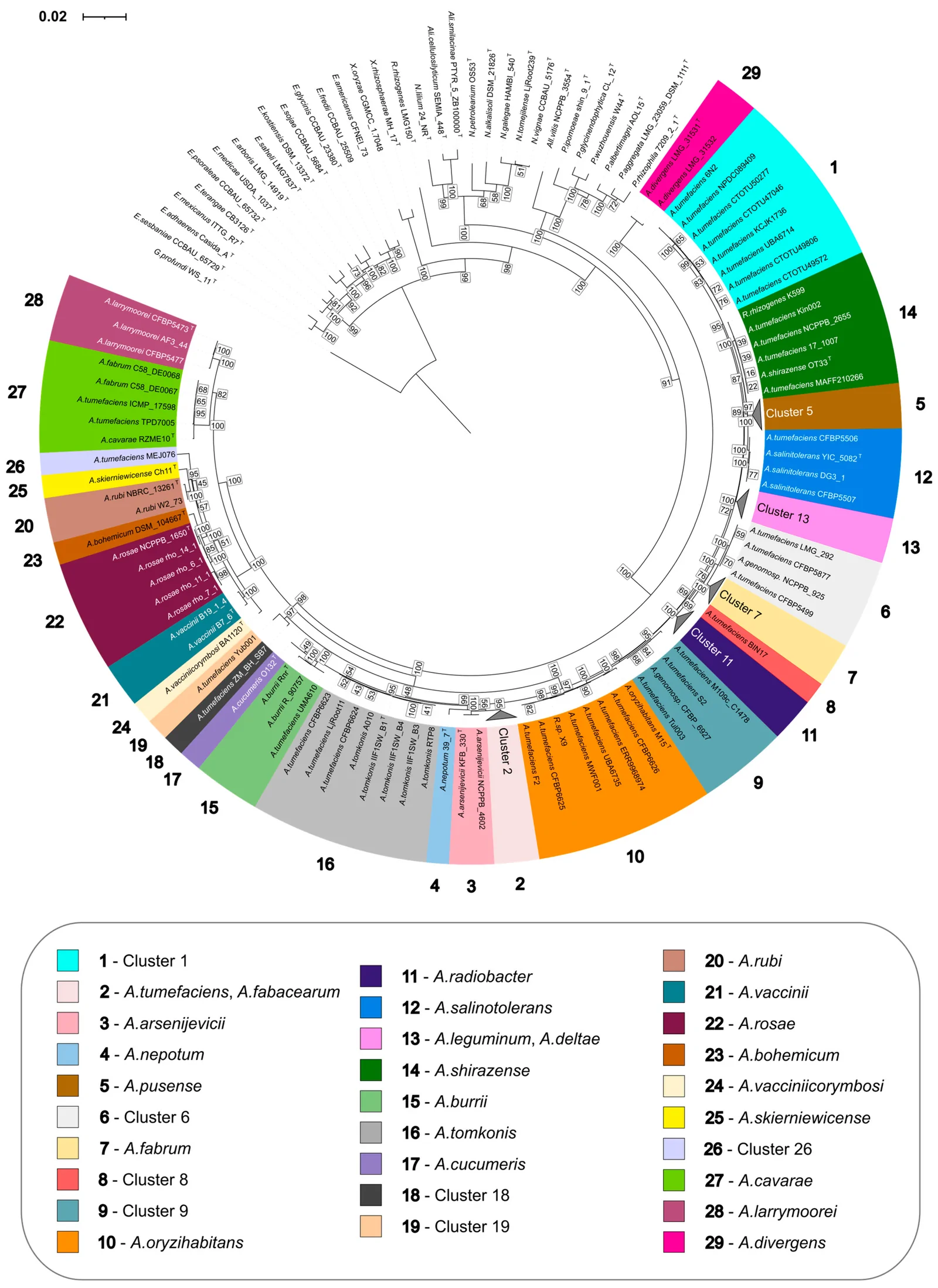
Maximum-likelihood phylogenetic tree inferred from 120 conserved bacterial marker proteins (GTDB-Tk) for 432 genomes of the *Agrobacterium* complex and related taxa. The tree was rooted using *G. profundi* WS-11^T^ as the outgroup. Numbers at nodes indicate ultrafast bootstrap support values (%). Cluster designations are shown around the tree perimeter.

All 20 *Agrobacterium* ANI 95% clusters represented by two or more genomes were recovered as monophyletic groups in the GTDB-marker ML topology (Figure 6) and bootstrap consensus, with ultrafast bootstrap support of 95–100%; nine clusters contained single genomes. Cluster 13 comprised 56 genomes, including strains 17_1850B_1b and 17_1850_1d, and received 100% support. Contracting internal branches shorter than 0.0002 substitutions per site preserved the boundaries of all 20 multi-genome clusters. Filtering consensus branches with support below 95% also preserved these boundaries, although some within-cluster relationships became unresolved.

### 2.4. Phylogenomics Based on Ribosomal Proteins

**Figure 7:**
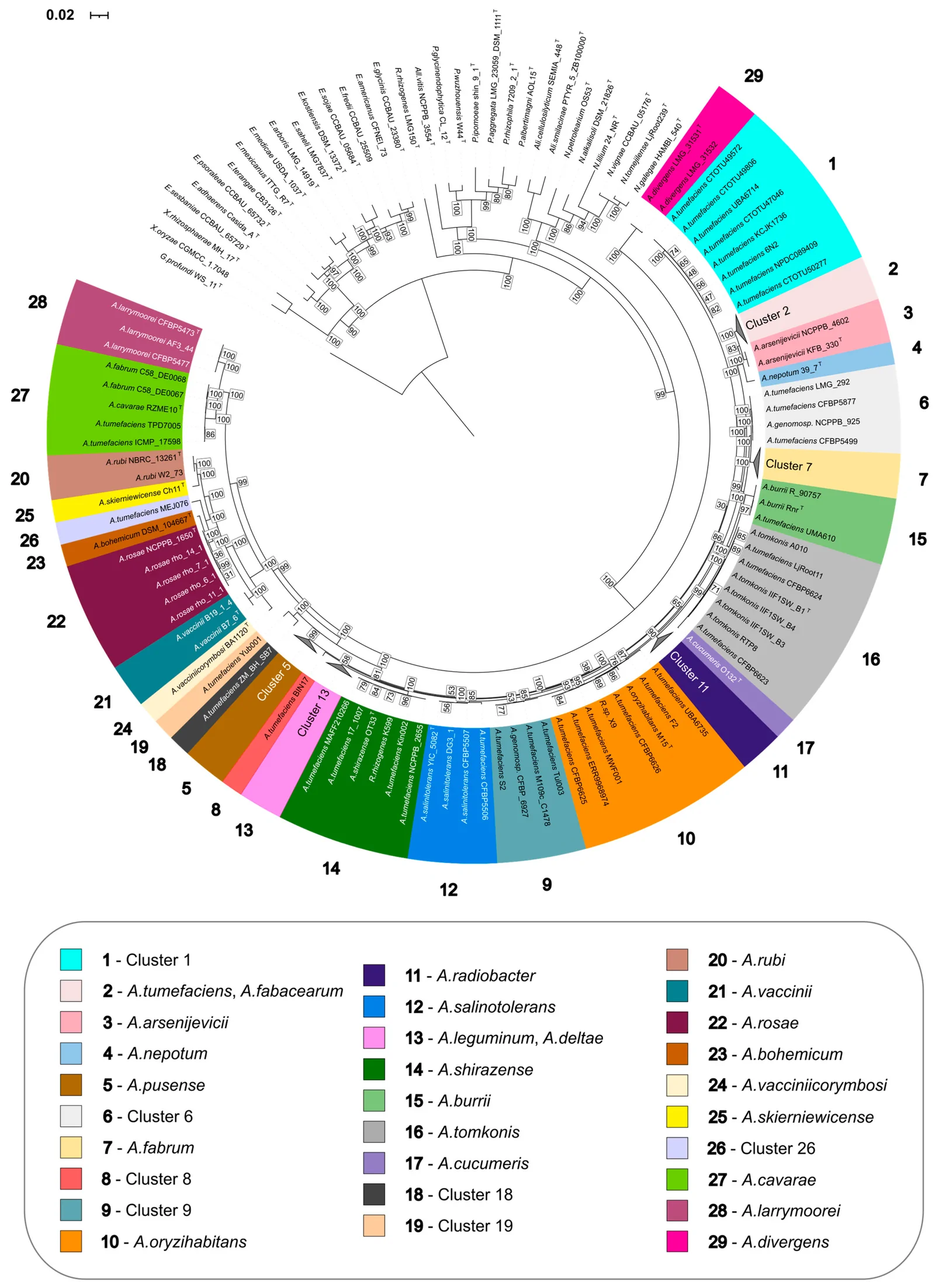
Maximum-likelihood phylogenetic tree inferred from concatenated sequences of 42 ribosomal proteins for 432 genomes of the *Agrobacterium* complex and related taxa. The tree was rooted using *G. profundi* WS-11^T^ as the outgroup. Numbers at nodes indicate ultrafast bootstrap support values (%). Cluster designations are shown around the tree perimeter.

A second phylogeny was inferred from concatenated sequences of 42 ribosomal proteins (Figure 7).

The alignment for the ribosomal protein phylogeny contained 432 taxa and 6525 amino acid positions. The final edge-linked proportional scheme comprised three partitions containing 4328, 1512, and 685 sites. ModelFinder selected Q.INSECT+F+R4 for the first and second partitions and Q.PLANT+I+G4 for the third under BIC; parameters were estimated separately for each partition. Branch support was assessed with 1000 ultrafast bootstrap replicates. The maximum-likelihood tree had a log-likelihood of −65,755.66 and contained 109 near-zero internal branches (<0.0002 substitutions per site). The bootstrap consensus tree had a log-likelihood of −65,761.71 and a Robinson–Foulds distance of 75 from the ML topology.

All 400 *Agrobacterium* genome records formed a clade with 100% ultrafast bootstrap support. The bootstrap consensus recovered all 20 multi-genome ANI 95% clusters, but support varied among them: Clusters 5 and 22 received 99%, Clusters 9 and 12 received 85%, Cluster 10 received 87%, Cluster 11 received 90%, and Cluster 13 received 58%; the other 13 multi-genome clusters received 100%. The nine singleton clusters do not provide a test of within-cluster monophyly.

Cluster 13 differed between the ribosomal-protein ML topology and bootstrap consensus. In the consensus, all 56 genomes formed a group with 58% support, with 54 genomes forming a subgroup supported at 96%. Strains 17_1850B_1b and 17_1850_1d formed a separate pair within that group. In the ML topology, Cluster 13 was not monophyletic: the smallest clade containing its representatives also contained the six genomes assigned to ANI Cluster 14. This local discordance was absent from the GTDB-marker phylogeny, in which Cluster 13 received 100% support.

Contracting near-zero branches preserved the consensus boundaries of the multi-genome ANI 95% clusters, while reducing resolution within some clusters. Filtering consensus branches with support below 95% removed the boundaries of Clusters 9–13, including the weakly supported Cluster 13 group. These relationships were therefore treated as unresolved rather than as evidence for revising the ANI-based assignments.

### 2.5. Pan-Genome and Metabolic Analysis

**Figure 8:**
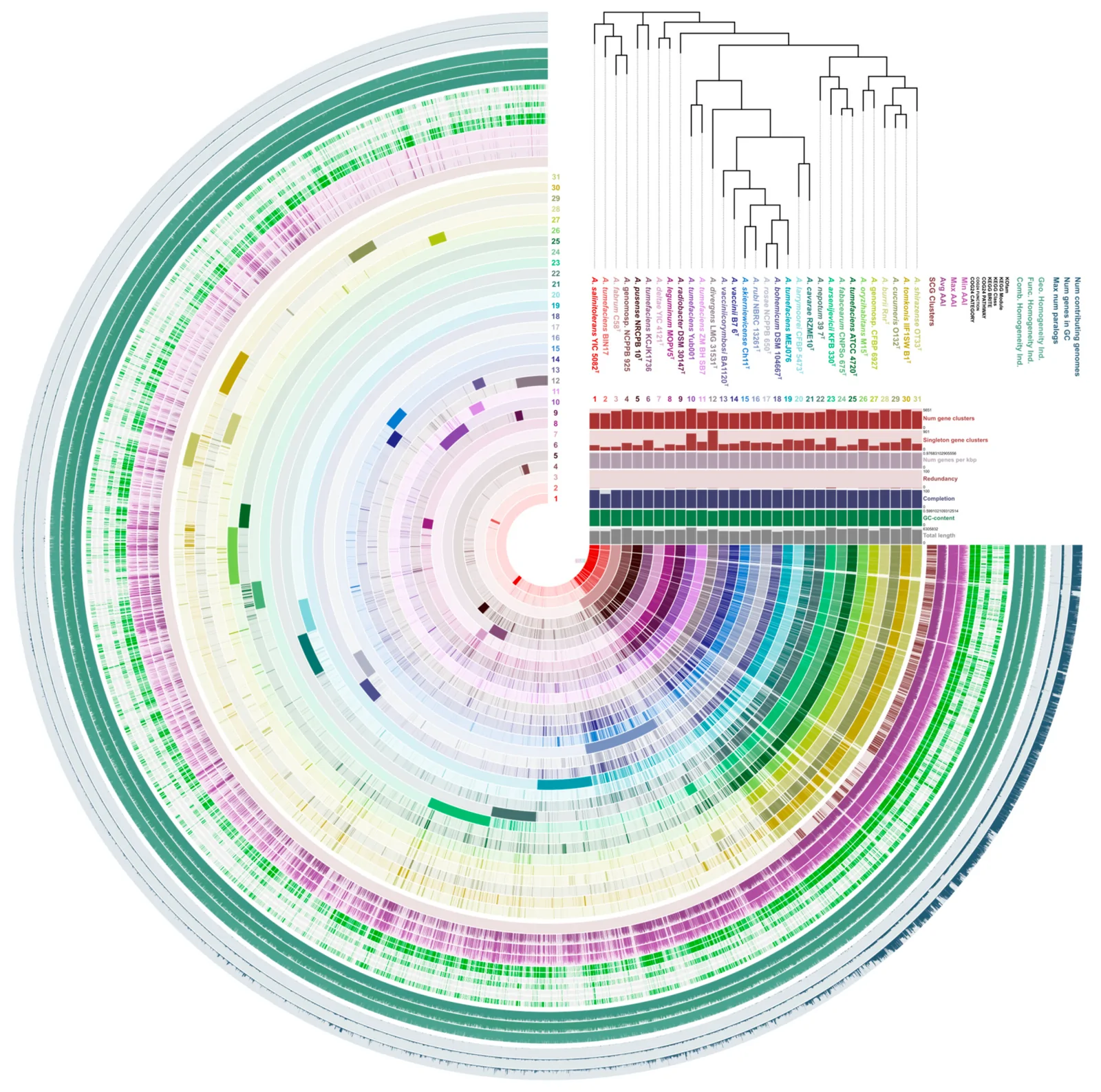
Pan-genome map of 31 representative *Agrobacterium* genomes. Concentric rings show gene-cluster distributions; core, soft-core, shell, cloud, and private fractions are indicated. Outer rings show COG24, KEGG, and KOfam annotation coverage.

Pan-genome analysis of 31 representatives of the major *Agrobacterium* lineages yielded 22,177 gene clusters (Figure 8). The map was displayed in forced synteny mode relative to “*A. fabrum*” C58, with genomes ordered by a single-copy core-gene phylogeny.

The strict core, present in all 31 genomes, comprised 2117 gene clusters. Another 301 clusters occurred in 30 genomes and were classified as soft core. Together, these fractions accounted for 2418 clusters, or 10.9% of the pan-genome.

The shell fraction contained 3783 clusters present in 5–29 genomes, and the cloud fraction contained 4527 clusters present in 2–4 genomes. The private fraction comprised 11,449 clusters found in only one representative (51.6% of the pan-genome). The three variable fractions together accounted for 89.1% of all gene clusters.

COG24 category or function assignments were available for 13,424 gene clusters (60.5%). COG24 pathway annotations were assigned to 2456 clusters and KOfam annotations to 8046; 1347 clusters had KEGG Class and KEGG Module annotations.

**Figure 9:**
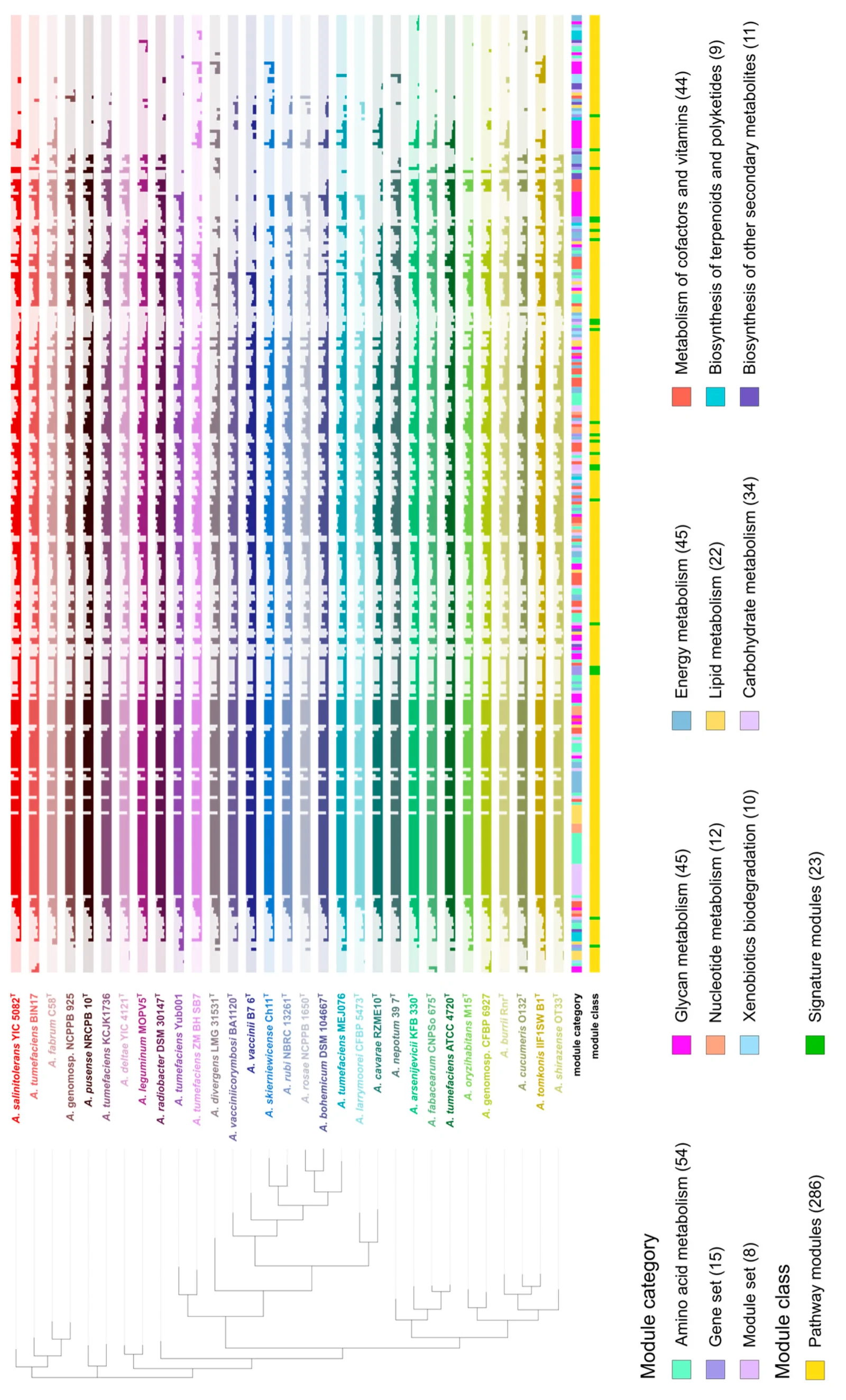
KEGG module completeness in 31 representative *Agrobacterium* genomes. Rows represent genomes and columns represent KEGG modules, including metabolic and other functional modules. Color intensity indicates reconstruction completeness from 0 to 1. Genomes are ordered according to a phylogenetic tree reconstructed from single-copy core genes (SCGs); the dendrogram alongside the heatmap represents this SCG-based phylogeny.

KEGG module completeness was evaluated across 400 *Agrobacterium* genome records in 29 clusters defined at 95% ANI. The analysis included 342 modules, with presence defined as pathwise completeness ≥0.75. Figure 9 displays the 31 representative genomes; prevalence estimates and statistical comparisons use the full 400-record set (Supplementary Table S4).

Of the 342 modules evaluated, 148 reached the presence threshold in at least one genome, whereas 194 did not reach the threshold in any retained genome. Ninety-one modules reached the threshold in at least 95% of genomes, including 58 detected in all 400 genomes. These counts refer to threshold-based module reconstruction rather than complete reconstruction of every module: only 26 modules had completeness equal to 1.00 in all genomes.

The cluster-association analysis identified 114 associations meeting both the adjusted *p*-value and prevalence-difference criteria, involving 46 unique modules. Of these associations, 53 represented higher prevalence within the respective cluster and 61 represented lower prevalence. The two largest clusters were Cluster 2 (120 genomes) and Cluster 11 (105 genomes).

Cluster 2 showed higher prevalence of pantothenate biosynthesis (M00913; 96.7% (116/120) versus 11.1% (31/280) outside the cluster), GDP-L-fucose biosynthesis (M01016; 100.0% (120/120) versus 26.8% (75/280)), DMSP degradation (M00991; 78.3% (94/120) versus 13.9% (39/280)), and siroheme biosynthesis (M00846; 100.0% (120/120) versus 43.9% (123/280)). In contrast, UDP-Galf biosynthesis (M01004) was detected in 3.3% (4/120) versus 81.1% (227/280), while denitrification (M00529) and oxygen-independent ubiquinone biosynthesis (M00989) were each detected in 23.3% (28/120) versus 79.6% (223/280).

The predicted AdeABC efflux module (M00649) reached the completeness threshold in 97.1% (102/105) versus 9.8% (29/295) in Cluster 11 and outside the cluster, respectively. Cluster 11 also showed lower prevalence of siroheme biosynthesis (M00846; 4.8% (5/105) versus 80.7% (238/295)) and ectoine degradation (M00919; 25.7% (27/105) versus 86.1% (254/295)). These results describe cluster-associated differences in predicted functions; reconstruction of an efflux module does not establish an antibiotic-resistance phenotype.

Phenylacetate degradation (M00878) reached the threshold in all genomes of Clusters 10 (8/8), 9 (4/4), and 28 (3/3), compared with 2.3% (9/392), 3.3% (13/396), and 3.5% (14/397) outside the respective clusters. All three associations met the specified criteria, although the estimates for Clusters 9 and 28 were based on small sample sizes.

Purine degradation (M00546) was more prevalent in Clusters 1, 5, and 7, with within-cluster versus outside-cluster prevalence of 100.0% (8/8) versus 30.9% (121/392), 93.3% (14/15) versus 29.9% (115/385), and 89.7% (26/29) versus 27.8% (103/371), respectively. Cluster 7 also showed higher prevalence of GDP-L-fucose biosynthesis (M01016; 96.6% (28/29) versus 45.0% (167/371)). The lower prevalence of methionine degradation (M00035; 34.5% (10/29) versus 65.8% (244/371)) did not remain significant after correction (*q* = 0.079) and was not retained as a cluster association.

Trehalose biosynthesis (M00565) was enriched in Cluster 5 (93.3% (14/15) versus 12.7% (49/385)) and reached 100% prevalence in Clusters 1 (8/8), 6 (4/4), 22 (5/5), and 27 (5/5); each of these associations met the specified criteria. Cytochrome bd ubiquinol oxidase (M00153) did not reach the threshold in any genome of Clusters 1, 27, or 28, compared with 94.9% (372/392), 94.2% (372/395), and 93.7% (372/397) outside these clusters, respectively. The three biotin-biosynthesis modules M00123, M00577, and M00950 were detected in 20.0% (1/5) of Cluster 22 genomes and in none of the five Cluster 27 genomes, versus 94.7% (374/395) and 94.9% (375/395) outside the respective clusters. Assimilatory sulfate reduction (M00176) was also depleted in Cluster 27 (0/5 versus 374/395), but did not meet the association criteria in Cluster 22. These findings should be interpreted in light of the small cluster sizes and the overlap between alternative modules describing related biosynthetic functions.

Six modules reached the presence threshold in only one genome: GABA biosynthesis from putrescine (M00135) in *A. tumefaciens* G-8; the reductive pentose phosphate cycle/Calvin cycle (M00165) in *A. deltae* RV3; archaeal-type thiamine biosynthesis (M00896) in *A. tumefaciens* UBA6735; heme biosynthesis via coproporphyrin III (M00926) in *Agrobacterium* sp. El2ro-1b; GDP-D-Rha4NAc biosynthesis (M01020) in *A. pusense* CFBP5875; and UDP-ManNAc3NAcA biosynthesis (M01035) in *A. divergens* LMG 31532. Their completeness values were 0.750, 0.818, 0.800, 0.778, 1.000, and 1.000, respectively. Thus, four of these singleton observations were partial reconstructions meeting the chosen threshold. They should not be interpreted as experimentally established phenotypes or cluster-wide functional traits.

### 2.6. Ti/Ri Plasmid-Associated Signatures and Resistance-Related Genes

Across the 432 retained genome records, 174 (40.3%) had a Ti-associated marker profile, seven (1.6%) had an Ri-associated profile, six (1.4%) had a partial vir-associated profile, and 245 (56.7%) had no detected signature (Supplementary Table S5). Within the 400-record *Agrobacterium* set, the corresponding counts were 173, six, six, and 215. The seven Ri-associated records were deposited as *R. rhizogenes* K599 and LMG 150^T^; *A. tumefaciens* NCPPB 2655, MAFF 210266, and MAFF 301724; *A. cucumeris* O132^T^; and *A. tomkonis* A010. These categories describe detected marker combinations and do not establish the presence of complete plasmids or experimentally demonstrated pathogenicity.

AMRFinderPlus reported 347 hits across 249 of the 432 retained genome records: 298 hits were assigned to AMR and 49 to STRESS (Supplementary Table S6). The output contained six named AMR classes, two named stress-related classes, and an unassigned class (NA). Gene prevalence was calculated from the number of distinct positive genome records rather than the number of hits. *catB1* was detected in 243 genomes (56.25%; 247 hits) and *tet(30)* in 29 genomes (6.7%; 29 hits). Among the 400 *Agrobacterium* records, their prevalence was 60.75% and 7.25%, respectively.

Less frequent AMR-associated hits comprised *aac(3)-Ia* (three genomes; 0.69%), *aph(3′′)-Ib* and *aph(6)-Id* (five genomes each; 1.16%), *aph(3′)-IIa* (two genomes; 0.46%), and *aph(6)-Ic*, *blaTEM-116*, *ble*, *sul1*, *sul2*, and *tet(C)* (one genome each; 0.23%). At least one mer-family hit was detected in six genomes (1.39%); *qacEdelta1* occurred in one genome (0.23%), and *yfdX1* and *yfdX2* each occurred in two genomes (0.46%). No hits assigned to *aadA*, *aph(3′)-Ia*, *tet(A)*, *qacE*, or *klaC* were present in the retained dataset.

The six genomes with partial vir-associated profiles carried *catB1*, with *tet(30)* detected in three, *aac(3)-Ia* in three, and *tet(C)* in one. The *blaTEM-116*, *aph(3′)-IIa*, *aph(6)-Ic*, and *ble* hits occurred in Ti-associated records, whereas *sul1*, *sul2*, and *qacEdelta1* occurred in records with no detected Ti/Ri-associated signature. mer-family and yfdX-family hits likewise occurred in the no-signature category. These observations describe genomic co-occurrence and do not establish physical linkage on the same replicon, mobility, or a causal association between marker-profile category and resistance-gene diversity.

## 3. Discussion

The clearest taxonomic discordance in this dataset concerns the use of the name *A. tumefaciens* for genomes belonging to several distinct lineages. Type-strain anchoring separates the lineage containing ATCC 4720^T^ from genomes grouped with *A. radiobacter*, *A. pusense*, and “*A. fabrum*”/G8. The placement of TPD7005 and ICMP 17598 with *A. cavarae*, and of MEJ076 in a separate lineage, extends this discrepancy beyond the principal *A. tumefaciens*/*A. radiobacter* groups. These assignments matter for comparative biology: pooling genomes by deposited name can combine between-lineage differences with variation within a lineage.

ANI clustering and conserved-protein phylogenies recovered broadly consistent major groups (Supplementary Table S7), but agreement depended on the topology considered. All multi-genome ANI 95% groups were monophyletic in the GTDB-marker phylogeny. The ribosomal-protein bootstrap consensus recovered the same groups, although Cluster 13 received only 58% support and was not monophyletic in the RP ML topology. This local discordance limits conclusions about the placement of boundary strains and does not by itself justify changing their ANI-based assignments. These analyses identify cases for taxonomic review rather than constituting a formal nomenclatural revision.

Threshold-dependent clustering requires particular care near species boundaries. We used 95% ANI for the main presentation and 96% to examine changes in membership, following the reference range used in bacterial taxonomy [9,14]. The KDE crossover near 94% is a descriptive feature of this dataset, influenced by sampling and the definition of comparison groups. It does not independently validate a species cutoff.

The possible conspecificity of *A. fabacearum* and *A. tumefaciens* has already been supported by comparative genomic analyses [16]. In our dataset, their type strains, CNPSo 675^T^ and ATCC 4720^T^, belonged to the same ANI cluster at both 95% and 96% with a pairwise ANI of 98.15% (calculated using FastANI). Their dDDH estimate, calculated using GGDC formula 2 (GBDP d4), was 86.0% (95% CI, 83.4–88.3%; Supplementary Table S2), above the conventional 70% species boundary [17]. This provides additional support for conspecificity. The possible treatment of *A. fabacearum* as a later heterotypic synonym of *A. tumefaciens* warrants formal taxonomic evaluation.

For *A. leguminum* and *A. deltae*, the type strains MOPV5^T^ and YIC 4121^T^ grouped together at 95% ANI but separated at 96% with a pairwise ANI of 95.97%. Their formula 2/d4 dDDH estimate was 66.7% (95% CI, 63.7–69.5%), below the conventional species boundary [17]. Vargas Ribera et al. previously suggested conspecificity using MLSA, whole-genome phylogeny, and ANIb; they referred to *A. deltae* as “*A. deltaense*” [8]. Our dDDH result supports retaining the distinction between these taxa. Membership in the same 95% cluster alone is insufficient to infer conspecificity or the exact pairwise ANI between the type strains.

The splitting of the *A. rubi* and *A. vaccinii* type-strain-containing clusters at 96% has a different interpretation. It identifies threshold-sensitive comparisons, but does not establish intraspecific variation: the non-type genomes may also be misidentified. Direct comparisons with the type strains and an audit of strain identities are needed before either explanation can be preferred.

At the genus level, the main evidence is the recovery of an *Agrobacterium* clade with 100% support in both phylogenies. Full-proteome AAI clustering is consistent with this grouping across 74–76%; *A. divergens* separates at 77%, and further fragmentation occurs at 79%. We therefore treat 76% as a dataset-specific operational cutoff, consistent with the value reported by van Lill et al. [15]. It should be distinguished from the approximately 86% core-proteome AAI criterion used by Kuzmanović et al., which is calculated from a different protein set [10].

Recent revisions of *Rhizobiaceae* further emphasize the importance of type-strain-centered phylogenomics for genus-level taxonomy [10,15]. Some strains of particular phytopathological relevance have subsequently been reassigned to newly circumscribed genera or species. Where historically established names were retained in the present dataset for comparison with earlier literature, their current taxonomic placement should be distinguished explicitly from the deposited or legacy designation. The present analysis provides a focused assessment of *Agrobacterium* within this broader taxonomic framework.

The five highlighted candidate species-level lineages (Clusters 1, 8, 18, 19, and 26) include groups recognized in earlier work. Cluster 1 contains UBA6714, 6N2, and KCJK1736, previously assigned to candidate genomospecies G21, whereas BIN17 was included in candidate G24 [8]. These groups represent priorities for taxonomic assessment rather than five newly established species. Clusters containing deposited genomospecies designations and reference strains of species proposed without valid publication should be distinguished from this set.

Gene-content variation was extensive among the 31 pan-genome representatives: core and soft-core clusters accounted for 10.9% of all clusters, and private clusters for 51.6%. These fractions describe the combined pan-genome across several species-level lineages. The broad taxonomic sampling provides one explanation for the large private fraction and limits its use as evidence for pan-genome openness. The origins and genomic locations of these variable clusters remain to be established.

The functional analysis addresses a related question at a different scale. Across the 400 *Agrobacterium* records, broadly shared metabolic modules coexisted with lineage-associated differences in predicted cofactor, sulfur and nitrogen metabolism, transport, and substrate degradation. Thus, the taxonomic structure contains information about functional variation. The enrichment and depletion patterns also show why a species name or cluster label should not be treated as a complete description of a genome’s functional repertoire.

The strongest prevalence contrasts in the large clusters involved pantothenate biosynthesis in Cluster 2 and the predicted AdeABC module in Cluster 11. Phenylacetate degradation was shared by several smaller groups, illustrating that an infrequently reconstructed module can occur in more than one lineage. These associations describe the sampled genomes; uneven cluster sizes and possible relatedness among isolates limit their extension to unsampled populations.

Module reconstruction indicates genomic potential rather than measured pathway activity. Intermediate completeness, differences in annotation, and missing sequences can affect presence calls.

Ti-associated profiles spanned several genomic lineages, whereas Ri-associated profiles were concentrated in a smaller part of this collection. This contrast adds a functional dimension to the type-strain-based grouping: related genomes can differ in the detected plasmid-associated profile, and similar profiles can occur in different lineages. Such variation is consistent with earlier studies of agrobacterial megaplasmids [13]. Ti plasmids carry functions for plant interaction and opine utilization [18,19]. Comparing their associated signatures with conserved-marker phylogenies therefore relates this aspect of functional variation to bacterial relatedness.

It should be noted that the observed pattern may reflect limitations of the current Ri-associated profile criteria. Rhizogenic agrobacteria have been reported from multiple *Agrobacterium* (genomo)species [8]. The limited number of Ri-associated profiles detected here may therefore reflect both the sampled collection and the marker-screening criteria; it should not be interpreted as the full taxonomic distribution of rhizogenic strains.

More broadly, published comparisons have shown that virulence plasmid relationships can differ from those of their bacterial hosts [3]. The distribution observed here reinforces the value of assessing taxonomic identity and Ti/Ri-associated signatures separately. The first describes genomic relatedness, while the second captures variation in a functionally relevant set of genes. A partial marker profile does not establish the presence of a structurally incomplete plasmid, and a genome with no detected signature cannot be classified as plasmid-free from this screen alone. Therefore, conclusions regarding the presence or absence of plasmids should be drawn cautiously when based solely on genome assemblies.

*catB1* was the most frequently detected resistance-related gene, whereas *tet(30)* was less common. Additional, less frequent hits further expanded the predicted resistance- and stress-related repertoires observed across the dataset. Their distribution provides a basis for comparing predicted resistance-related repertoires, while the corresponding phenotypes remain untested in this study.

Experimental characterization of AcrABR [20] and MacAB [21] in individual agrobacterial strains illustrates the functional relevance of transport-related genes. In the present collection, gene detection and co-occurrence identify patterns for further comparison; genomic context would be needed to establish the replicon locations of the detected genes and evaluate their potential mobility.

## 4. Materials and Methods

### 4.1. Genome Sampling

The initial dataset was assembled from NCBI on 11 August 2026 using the search term “*Agrobacterium tumefaciens* complex”. Additional related genomes were selected using a BLASTn search based on *rpoB* with default parameters and by including genomes of *Rhizobiaceae* that were used in the phylogeny of the core genome by Kuzmanović et al. [10]. Nomenclatural reference strains were selected using LPSN [11]. The initial set comprised 483 genome records. The names “*A. fabrum*”, “*A. bohemicum*”, and “*A. oryzihabitans*” are retained in quotation marks because they are not validly published. For these names, reference to a proposed type strain does not imply valid publication.

### 4.2. Genome Quality Control and Filtering

Genome completeness and contamination were estimated using CheckM2 v1.0.2. The stated quality criteria were completeness ≥95% and contamination ≤5%.

Of the initial 483 genome records, 18 failed the quality criteria: 13 because of completeness <95%, four because of contamination >5%, and one because of both criteria. Nineteen additional records were excluded as duplicate or redundant records. Twelve further records represented laboratory-derived strains or experimentally selected mutants: LBA4213, LBA4404, GV2260, EHA105, GV3101(pMP90), LBA645, NT1RE, A136, Arqua, ARqua1, ATCC 31749, and CGMCC 11546. These records were excluded from all downstream analyses. Redundant EHA105 and ARqua1 assemblies already removed during deduplication were also annotated as laboratory-derived in Supplementary Table S8. The IRBG74 record was excluded because the published sequencing study used a culture marked with mTn5ssgusA20 [22]. F4 was excluded separately as a conservative provenance-related decision; it was not classified as a confirmed laboratory-derived strain [23]. Thus, 14 additional records were removed after quality filtering and deduplication, leaving 432 records, including 400 assigned to *Agrobacterium* and 32 comparative records. Supplementary Table S8 provides accession identifiers, quality estimates, retention status and exclusion reasons.

### 4.3. Genome-Based Taxonomy Using TYGS

Genome-based taxonomic comparisons were performed with TYGS on 25 August 2026 for 31 type-strain and selected non-type-strain genomes [11,24,25]. Nomenclatural information was obtained from LPSN [11,25].

TYGS selected closely related type strains using MASH genome comparisons [26] and 16S rRNA gene searches. The latter used RNAmmer extraction [27], BLAST comparisons [28], and GBDP distances calculated with the coverage algorithm and formula d5 [17].

Pairwise genome comparisons were conducted using GBDP under the trimming algorithm and distance formula d5, with 100 distance replicates per comparison. dDDH values and confidence intervals were calculated using GGDC 4.0 with recommended settings [11,17]. For species-level interpretation, dDDH values obtained with GGDC formula 2 (GBDP d4; identities/HSP length) were used. Values obtained with the other GGDC formulas were retained in Supplementary Table S2 for completeness.

The TYGS report also included a balanced minimum-evolution tree inferred with FASTME 2.1.6.1 and SPR postprocessing [29], with 100 pseudo-bootstrap replicates, midpoint rooting [30], and PhyD3 visualization [31].

TYGS used dDDH thresholds of 70% for species clustering and 79% for subspecies clustering [24,32]. Species-level interpretation in this study used the formula 2/d4, with estimates reported in Supplementary Table S2.

### 4.4. Average Nucleotide Identity (ANI)

Pairwise ANI was calculated with ANIclustermap v2.0.1 (https://github.com/moshi4/ANIclustermap (accessed on 19 January 2026)) using FastANI v1.34 and default parameters [33]. The 432 × 432 matrix was subjected to UPGMA clustering. Cluster membership was compared at 95% and 96% ANI, with 95% used for the main presentation and type-strain assignments considered separately from the numerical cluster labels.

For descriptive analysis of the ANI distribution, pairwise values were extracted from the lower triangle of the matrix, excluding self-comparisons and restricting the set to the 400 *Agrobacterium* records. Gaussian kernel density estimation was performed with scipy.stats.gaussian_kde. The plotted distributions and the estimated KDE crossover were compared with the 95% and 96% clustering thresholds; the KDE crossover was not treated as an independently estimated species boundary.

### 4.5. Average Amino Acid Identity (AAI)

Pairwise AAI, standard deviation of AAI, and the proportion of shared orthologs for 432 genomes were calculated using CompareM v0.1.2 (https://github.com/dparks1134/CompareM (accessed on 28 June 2026)) with default parameters. The CompareM output was converted into three 432 × 432 square matrices (mean AAI, standard deviation, and percentage of orthologs) using Python v3.11 (pandas library). For visualization of the mean AAI matrix, a heatmap with hierarchical clustering (UPGMA) was constructed based on the distance 1-AAI/100; clustering and visualization were performed in Python v3.11 using matplotlib, seaborn, and scipy libraries. The heatmap color scale for AAI was defined using discrete thresholds to reflect genus-level differentiation.

AAI clustering was compared at 65%, 74%, 75%, 76%, 77%, 78%, and 79%. The operational cutoff was selected as the highest tested value retaining the sampled *Agrobacterium* genomes in one cluster while separating the included neighboring groups. KDE plots were used to describe intra-genus and inter-genus comparisons, with genus categories based on the assignments used in this study. Their intersection was treated as a descriptive crossover. Calculations used Python v3.11, scipy, and numpy.

### 4.6. GTDB-Tk Classification and Marker Phylogeny

Genome taxonomy was assigned using GTDB-Tk v2.7.2 and the Genome Taxonomy Database release R232 [34,35]. The standard workflow was applied to each genome, comprising identification of a set of 120 conserved bacterial marker genes, their multiple alignment against the GTDB reference sequences, and placement on the reference tree followed by taxonomic classification.

A separate maximum-likelihood phylogeny was inferred in IQ-TREE v3.0.1 from the GTDB-Tk marker alignment of 5010 amino acid positions [36]. ModelFinder selected Q.INSECT+F+I+R4 by BIC [37]. Branch support was assessed with 1000 ultrafast bootstrap replicates using random seed 24,677. The tree was rooted for display with *Georhizobium profundi* WS-11^T^ (GCA_003952725.1) and visualized in iTOL [38]. ANI 95% cluster membership was mapped onto the ML and bootstrap-consensus topologies. As post hoc sensitivity checks, internal branches shorter than 0.0002 substitutions per site were contracted, and consensus branches with ultrafast bootstrap support below 95% were filtered separately. These operations were used to assess retention of cluster boundaries, not to alter taxonomic assignments.

### 4.7. Phylogenomic Analysis of Ribosomal Proteins

Conserved ribosomal proteins were identified using UBCG2 v2.0, which extracts a set of 81 bacterial core genes. From this output, 42 ribosomal protein genes belonging to the *rpl*, *rpm*, and *rps* families were selected for subsequent phylogenetic analysis [39]. For each of the 42 proteins, multiple amino-acid sequence alignment was performed in Geneious v2025.2.2 (https://www.geneious.com (accessed on 18 December 2025)) using MAFFT v7.505 (L-INS-i algorithm), the BLOSUM30 scoring matrix, a gap-open penalty of 1.53, and an offset value of 0.123 [40]. The aligned sequences were concatenated into a single supermatrix containing 432 taxa and 6525 amino-acid sites. Partitioning according to the original gene boundaries was specified using AMAS v1.00 [41].

Maximum-likelihood inference used IQ-TREE v3.0.1 with ModelFinder and BIC-based model selection. The final edge-linked proportional scheme contained three partitions: Q.INSECT+F+R4 for partitions of 4328 and 1512 sites, and Q.PLANT+I+G4 for the remaining 685 sites. Each partition had separately estimated model parameters. Branch support was assessed with 1000 ultrafast bootstrap replicates using random seed 669,681. The tree was rooted for display with *G. profundi* WS-11^T^ and visualized in iTOL. Cluster-boundary sensitivity to short-branch contraction and support filtering was assessed as described for the GTDB-marker phylogeny.

### 4.8. Pan-Genome and Metabolic Analysis

Pan-genome and metabolic analyses used anvi’o v9 [42]. The pan-genome was calculated for 31 representatives of the major *Agrobacterium* lineages. Metabolic module prevalence and cluster associations were analyzed across all 400 *Agrobacterium* records, while the metabolic heatmap displayed the same 31 representatives.

Contigs databases for each genome were generated using anvi-gen-contigs-database based on reformatted FASTA files. For each genome, the original GenBank annotation file (deposited alongside the assembly) was used as the source of gene calls; no de novo gene prediction was performed. External genomes were then aggregated into a single genomes storage using the anvi-gen-genomes-storage command with the --gene-caller “GenBank” parameter, so that the original annotation was retained consistently across all genomes.

Pan-genome analysis was conducted using anvi-pan-genome with the following parameters: --minbit 0, --min-percent-identity 30, --mcl-inflation 2, --use-ncbi-blast, and --num-threads 16. Pan-genome visualization was performed using anvi-display-pan. For the representative-genome analysis, genomes were ordered according to a phylogenetic tree reconstructed from single-copy core genes (SCGs). The resulting SCG-based phylogeny was used to define the genome order displayed alongside the KEGG module-completeness heatmap. The tree was constructed with anvi-gen-phylogenomic-tree using the --just-do-it flag. The tree was imported into the database for layer ordering using anvi-import-misc-data with the -t layer_orders flag.

Functional annotation used COG24 and KEGG [43,44,45]. KEGG Ortholog assignments were obtained with anvi-run-kegg-kofams, and module completeness matrices with anvi-estimate-metabolism (--matrix-format). The matrices were visualized with anvi-interactive in manual mode.

Module presence was defined as pathwise completeness ≥0.75. For each module and each cluster, prevalence was compared between genomes within that cluster and all remaining genomes using a two-sided Fisher’s exact test on a 2 × 2 presence/absence table. Benjamini–Hochberg correction was applied jointly across all 9918 tests (29 clusters × 342 modules). Associations were retained when the adjusted *p*-value (*q*) was <0.05 and the absolute prevalence difference was ≥0.20 (20 percentage points). No minimum cluster-size filter was applied. Wilson 95% confidence intervals were calculated for within-cluster and outside-cluster prevalence. Completeness distributions and genome-level module counts were also summarized. Sensitivity analyses repeated the entire testing procedure at completeness thresholds of 0.50 and 1.00, with a separate correction across 9918 tests for each threshold. These analyses describe associations in the sampled genomes and do not model phylogenetic non-independence.

### 4.9. Screening for Ti/Ri Plasmid-Associated Signatures

All 432 retained genome records were screened for Ti/Ri-associated marker combinations. Reference protein sequences were retrieved from UniProt for members of *Rhizobiaceae*. The marker set included proteins from the VirB, VirC, and VirD families (virulence machinery), RolB and RolC (Ri plasmid root locus proteins), as well as IaaH, IaaM, and Ipt/Tzs (phytohormone biosynthesis). Nucleotide sequences of annotated coding regions were searched against the reference protein sequences using BLASTx v2.17.0 [28,46]. A protein was considered present if the best BLASTx hit met the following criteria: E-value < 1 × 10^−5^, sequence identity > 50%, and query coverage > 50%.

Records were classified sequentially into mutually exclusive categories. Detection of both RolB and RolC defined an Ri-associated profile. Among the remaining records, detection of at least two markers from {IaaH, IaaM, Ipt/Tzs} defined a Ti-associated profile. Records meeting neither criterion but containing both VirB- and VirC-family hits were classified as partial vir-associated profiles; all others were classified as having no detected signature. The screen did not establish replicon localization, plasmid identity, structural completeness, or pathogenicity, and was not designed to characterize non-Ti/Ri accessory plasmids.

### 4.10. Detection of Antibiotic Resistance and Stress-Related Genes

All 432 genome records were screened with NCBI AMRFinderPlus v4.2.7 using nucleotide input, default search parameters, and the reference database dated 26 August 2026 [47]. Hits were summarized using the database’s core and plus scope categories, which do not denote the bacterial core and accessory genome. Gene prevalence was defined as the number of distinct genome records with at least one hit for the corresponding element symbol divided by 432; *Agrobacterium*-only prevalence used the 400-record subset. Multiple hits for the same symbol within one genome were counted separately as hits but only once for prevalence. Family-level frequencies were calculated from the union of positive genomes. Detection was interpreted as a predicted determinant or annotated stress-related element without phenotypic resistance testing.

## 5. Conclusions

Analysis of 432 genome records identified 29 *Agrobacterium* groups at the 95% ANI threshold and a genus-level clade supported by both conserved-protein phylogenies. All multi-genome ANI groups were monophyletic in the GTDB-marker phylogeny, while Cluster 13 showed local discordance between the ribosomal-protein ML topology and bootstrap consensus. Type-strain anchoring placed genomes deposited as *A. tumefaciens* in several lineages, demonstrating that deposited names alone can obscure species-level diversity.

Type-strain dDDH supported conspecificity of *A. fabacearum* and *A. tumefaciens* (86.0%) and the distinction between *A. leguminum* and *A. deltae* (66.7%). The latter pair clustered together at 95% ANI but separated at 96%, illustrating the need to combine numerical clustering with direct type-strain comparisons. Five highlighted candidate species-level lineages merit further taxonomic assessment, including groups overlapping candidate genomospecies G21 and G24.

Pan-genome analysis of 31 representatives and KEGG module reconstruction across 400 *Agrobacterium* records revealed extensive gene-content variation and lineage-associated differences in predicted functional potential. The full 432-record screen detected 174 Ti-associated, seven Ri-associated, and six partial vir-associated profiles; these signatures do not establish complete plasmids or pathogenicity. *catB1* and *tet(30)* occurred in 243 and 29 genomes, respectively, without phenotypic resistance testing. These findings support taxonomic reassessment while distinguishing genomic predictions from experimentally demonstrated functions.

## Figures and Tables

**Table 1:** *Agrobacterium* ANI clusters at 95% and 96%. Numbers in parentheses indicate genome records in the current analysis. Deposited names are retained to show taxonomic discordance. Names in quotation marks are not validly published; for these names, superscript T identifies the strain designated in the original proposal. genomosp.—designation for a genomic species (genomospecies) within the *Agrobacterium tumefaciens* complex that has not yet been formally named; it was used in the original genome records. Putative species-level lineage—an unresolved ANI-defined lineage lacking a nomenclatural type-strain representative; it may overlap a previously proposed candidate genomospecies. This designation does not constitute a formal species proposal.

Cluster (95%)	Cluster (96%)	Key Taxa	Notes
1 (8)	1 (8)	*A. tumefaciens* (CTOTU49572, CTOTU49806, CTOTU47046, UBA6714, CTOTU50277, KCJK1736, NPDC089409, 6N2)	Putative species-level lineage; overlaps candidate G21 (see Discussion)
2 (120)	2 (120)	*A. tumefaciens* ATCC 4720^T^, *A. fabacearum* CNPSo 675^T^, numerous *A. tumefaciens*, *A. fabacearum*	Largest *Agrobacterium* cluster; includes the type strains of *A. tumefaciens* and *A. fabacearum*
3 (2)	3 (2)	*A. arsenijevicii* KFB 330^T^, *A. arsenijevicii* NCPPB 4602	Species *A. arsenijevicii*
4 (1)	4 (1)	*A. nepotum* 39/7^T^	Species *A. nepotum*
5 (15)	5 (15)	*A. pusense* NRCPB10^T^, many *A. pusense*, *A. tumefaciens* (S33, UBA2572)	Species *A. pusense,* some *A. tumefaciens*
6 (4)	6 (4)	*A. tumefaciens* (LMG 292, CFBP 5877), *A.* genomosp. NCPPB 925, *A. tumefaciens* CFBP 5499	Includes the *A.* genomosp. NCPPB 925
7 (29)	7 (26) + 8 (3)	“*A. fabrum*” C58, many “*A. fabrum*” strains, *A. tumefaciens* A74a	At 96% split: “*A. fabrum*” PL3 S12_1, *A.* sp. El2ro-1b, and *A. tumefaciens* A74a in Cluster 8; the name “*A. fabrum*” has not yet been validly published.
8 (1)	9 (1)	*A. tumefaciens* BIN17	Putative species-level lineage; BIN17 previously included in candidate G24
9 (4)	10 (4)	*A. tumefaciens* Tul003, *A.* genomosp. CFBP 6927, *A. tumefaciens* S2, *A. tumefaciens* M109c_C1478	Includes the *A.* genomosp. CFBP 6927
10 (8)	11 (8)	“*A. oryzihabitans*” M15^T^, *A. tumefaciens* (ERR9968974, UBA6735, CFBP 6626, CFBP 6625, F2, MWF001), *R.* sp. X9	Includes the proposed type strain of “*A. oryzihabitans*”; name not validly published
11 (105)	12 (105)	*A. radiobacter* DSM 30147^T^, *A. tumefaciens*	Species *A. radiobacter*
12 (4)	13 (3) + 14 (1)	*A. salinitolerans* YIC 5082^T^, *A. salinitolerans* (CFBP 5507, DG3-1), *A. tumefaciens* CFBP 5506	At 96% split: *A. salinitolerans* in Cluster 13; *A. tumefaciens* in Cluster 14
13 (56)	15 (7) + 16 (39) + 17 (10)	*A. leguminum* MOPV5^T^, *A. leguminum* (AT13, EL101, CFBP 4996), *A. deltae* YIC 4121^T^*, A*. *deltae* NCPPB 1641, *A.* sp. T29, many *A. tumefaciens*	At 96% split into three clusters: Cluster 17 includes type strain *A. leguminum* + 9 others; 7 strains in Cluster 15; 39 strains in Cluster 16
14 (6)	18 (6)	*A. shirazense* OT33^T^, *R. rhizogenes* K599, *A. tumefaciens* (Kin002, 17-1007, NCPPB 2655, MAFF210266)	Includes *R. rhizogenes*; includes the type strain of *A. shirazense*
15 (3)	19 (3)	*A. burrii* Rnr^T^, *A. burrii* R-90757, *A. tumefaciens* UMA610	Species *A. burrii*
16 (8)	20 (8)	*A. tomkonis* IIF1SW_B1^T^, *A. tomkonis* (RTP8, IIF1SW-B3, IIF1SW-B4, A010), *A. tumefaciens* (CFBP 6624, CFBP 6623, LjRoot11)	*A. tomkonis*
17 (1)	21 (1)	*A. cucumeris* O132^T^	Species *A. cucumeris*
18 (1)	22 (1)	*A. tumefaciens* ZM-BH-SB7	Putative species-level lineage
19 (1)	23 (1)	*A. tumefaciens* Yub001	Putative species-level lineage
20 (2)	24 (1) + 25 (1)	*A. rubi* NBRC 13261^T^, *A. rubi* W2/73	At 96% split: type strain in Cluster 25, W2/73 in Cluster 24
21 (2)	26 (1) + 27 (1)	*A. vaccinii* B7.6^T^, *A. vaccinii* B19.1.4	At 96% split: type strain in Cluster 27, B19.1.4 in Cluster 26
22 (5)	28 (5)	*A. rosae* NCPPB 1650^T^, *A. rosae* (rho-11.1, rho-7.1, rho-6.1, rho-14.1)	Species *A. rosae*
23 (1)	29 (1)	“*A. bohemicum*” DSM 104667^T^	“*A. bohemicum*” lineage; name not validly published
24 (1)	30 (1)	*A. vacciniicorymbosi* BA1120^T^	Species *A. vacciniicorymbosi*
25 (1)	31 (1)	*A. skierniewicense* Ch11^T^	Species *A. skierniewicense*
26 (1)	32 (1)	*A. tumefaciens* MEJ076	Putative species-level lineage
27 (5)	33 (5)	*A. cavarae* RZME10^T^, genomes deposited as “*A. fabrum*” C58 DE0068 and C58 DE0067, *A. tumefaciens* TPD7005 and ICMP 17598	Species *A. cavarae*
28 (3)	34 (3)	*A. larrymoorei* CFBP 5473^T^, *A. larrymoorei* CFBP 5477, AF3.44	Species *A. larrymoorei*
29 (2)	35 (2)	*A. divergens* LMG 31531^T^, LMG 31532	Species *A. divergens*

## Data Availability

This study analyzed publicly available genome assemblies and sequence data. Source accessions are listed in Supplementary Table S8. Computational workflows and intermediate analysis files are available from the corresponding authors upon reasonable request.

## Supplementary Materials

The following supporting information can be downloaded at https://www.mdpi.com/article/10.3390/ijms27209210/s1.

## Abbreviations

The following abbreviations are used in this manuscript:

AAI	average amino acid identity
AFLP	amplified fragment length polymorphism
ANI	average nucleotide identity
CpAAI	core-proteome AAI
DDH	DNA-DNA hybridization
dDDH	digital DNA-DNA hybridization
GTDB	Genome Taxonomy Database
KDE	kernel density estimation
KEGG	Kyoto Encyclopedia of Genes and Genomes
LPSN	List of Prokaryotic names with Standing in Nomenclature
MLSA	multilocus sequence analysis
NNI	nearest neighbor interchange algorithm
